# Early child stimulation, linear growth and neurodevelopment in low birth weight infants

**DOI:** 10.1186/s12887-022-03579-6

**Published:** 2022-10-08

**Authors:** Ravi Prakash Upadhyay, Sunita Taneja, Tor A. Strand, Halvor Sommerfelt, Mari Hysing, Sarmila Mazumder, Nita Bhandari, Jose Martines, Tarun Dua, Patricia Kariger, Rajiv Bahl

**Affiliations:** 1 Centre for Health Research and Development, Society for Applied Studies, 45, Kalu Sarai, New Delhi, 110016 India; 2 Department of Global Public Health and Primary Care, University of Bergen, Bergen, Norway; 3 Department of Research, Innlandet Hospital Trust, Lillehammer, Norway; 4 Centre for Intervention Science in Maternal and Child Health (CISMAC), Department of Global Public Health and Primary Care, University of Bergen, Bergen, Norway; 5 Cluster for Global Health, Division for Health Services, Norwegian Institute of Public Health, Oslo, Norway; 6 Department of Psychosocial Science, Faculty of Psychology, University of Bergen, Bergen, Norway; 7 Department of Mental Health and Substance Use, World Health Organization, Geneva, Switzerland; 8 Center for Effective Global Action (CEGA), School of Public Health, University of California, Berkeley, USA; 9 Department of Maternal, Newborn, Child and Adolescent Health, World Health Organization, Geneva, Switzerland

**Keywords:** Linear growth, Child stimulation, Neurodevelopment, Low birth weight, Infancy

## Abstract

**Background:**

Children with low birth weight (LBW) are at risk of linear growth faltering and developmental deficits. Evidence suggests that early child stimulation and care reflected as responsive caregiving and opportunities for learning can promote development. The current analysis aimed to measure the extent to which linear growth and early child stimulation modify each other’s association with neurodevelopmental outcomes among LBW infants.

**Methods:**

This is a secondary data analyses from a randomized controlled trial on the effect of community-initiated kangaroo mother care in LBW infants on their neurodevelopment at 12 months of corrected age. Bayley Scales of Infant and Toddler Development was used to assess cognitive, motor and language scores. Stimulation at home was assessed by the Pediatric Review of Children’s Environmental Support and Stimulation (PROCESS) tool. PROCESS scores were categorized into three groups: < Mean-1SD (*low stimulation)*; Mean ± 1 SD (*moderate stimulation)* and > mean + 1SD (*high stimulation)*.

**Results:**

A total of 516 infants were available for neurodevelopment assessments. Interactions were observed between length for age z-score (LAZ) and PROCESS score categories. In the low stimulation group, the adjusted regression coefficients for the association between LAZ and cognitive, motor and language scores were substantially higher than in the moderate and high stimulation group. Stimulation was positively associated with neurodevelopmental outcomes in both stunted and non-stunted infants; however, the association was twice as strong in stunted than in non-stunted.

**Conclusion:**

Moderate to high quality stimulation may alleviate the risk of sub-optimal development in LBW infants with linear growth deficits.

**Clinical trial registration:**

The primary trial whose data are analysed is registered at clinicaltrials.gov (https://clinicaltrials.gov/ct2/show/NCT02631343).

**Supplementary Information:**

The online version contains supplementary material available at 10.1186/s12887-022-03579-6.

## What is already known on this topic?

- Linear growth and quality of stimulation and nurturance are independently known to influence neurodevelopment, especially in children born with low birth weight (LBW).
- The extent to which growth and stimulation influence each other’s association with cognitive, motor and language scores is unknown.

## What this study adds?

- High quality stimulation and nurturance could protect LBW infants with growth deficits from poor development scores.
- Association of stimulation with neurodevelopmental outcomes was twice as strong in stunted than in non-stunted infants.

## Introduction

The first 1000 days i.e., from conception through age 24 months, are foundational for brain development [1]. Both adverse and positive experiences during this period may critically shape children’s developmental trajectories [2, 3]. Children born with low birth weight (LBW) are at risk of linear growth faltering, cognitive and motor deficits as well as lower academic performance and behavioural problems compared to their normal birth weight counterparts [4–7]. Linear growth faltering in the first 2 years of life has been shown to be negatively associated with cognitive performance in childhood [8, 9]. There is also strong evidence that a child’s positive home environment reflected as responsive caregiving and opportunities for early learning, can promote development [10–12].

Less is known on whether linear growth and quality of stimulation/responsive caregiving at home influence each other’s association with cognitive, motor and language scores. Using a sample of 513 infants from rural India, Black et al. showed that a nurturant home environment attenuated associations between linear growth and fine motor and receptive language development [13]. Similarly, another multicentre study from Burkina Faso, Ghana and Malawi did not detect significant association between linear growth faltering and child development in the context of a high-quality developmental stimulation [14]. A study from rural Vietnam noted a modest beneficial effect of early child development interventions on cognition among children with declining height-for-age Z-scores or those that were stunted [15]. These findings indicate that in the presence of an environment characterized by nurturance and learning opportunities, children with low length-for- age z score (LAZ) can acquire developmental skills at the same level as their peers. Contrasting these findings, recent studies from Malaysian and Jamaican infants found no significant influence of home environment quality on the association between LAZ status and cognitive outcomes [16, 17]. More evidence is required on the interactive effects of linear growth and home environment in relation to developmental outcomes, particularly for the vulnerable subset of LBW infants. Further, evidence is required on whether in a setting with socio-economic constraints, a moderate to high-quality home environment can protect LBW infants with growth deficits from poor development scores and whether there is a differential effect of stimulation on developmental outcomes based on whether the LBW infant is stunted or not. The present analysis was aimed at providing insights on these pertinent issues of global importance.

## Methods

### Study design and participants

This secondary data analysis was conducted using data from an individually randomized controlled trial (RCT) aimed to evaluate the effect of community-initiated Kangaroo Mother Care (ciKMC) on neurodevelopmental outcomes of infants born low birth weight at 12 months of corrected age (ClinicalTrials.gov identifier NCT02631343) [18]. The study was conducted in resource constrained settings of rural and semi-urban Haryana, North India. In this study population, ciKMC was not associated with the neurodevelopment measures at 12 months [18]. A total of 552 stable preterm or small for gestational age term infants identified within 72 hours of birth and weighing between 1500 and 2250 g were included in the trial and followed up till 12 months of age. In the primary trial, infants weighing between 1500 and 1800 g, as per the government recommendations, were initially referred to a health facility for evaluation. These infants were considered for inclusion only if the families refused to take the baby to the health facility, or if the baby was taken but the medical doctor/paediatrician did not recommend admission or if admission was done, it was for less than 72 hours [18]. The primary trial excluded infants who were unable to feed, had difficulty in breathing, had less than normal movements and those with gross congenital malformations. As this was a trial assessing the efficacy of Kangaroo Mother Care (KMC) initiated at home/community, those infants who had KMC initiated at the health facility were excluded [18].

Details of the trial have been published elsewhere [18, 19]. Ethical clearances for the primary trial were obtained from the Institutional Ethics Review Committee of Society for Applied Studies, New Delhi (SAS/ERC/KMC-GCC/2015), the World Health Organization (WHO) Ethics Review Committee, Geneva (ERC0002629) and the Regional Committee for Medical and Health Research Ethics in Norway. In the primary trial, written informed consent was obtained from all subjects and/or their legal guardian(s).

### Exposure and outcomes

Baseline information was collected on maternal and paternal age and education, birth order, parity and sex of the infant. Gestational age was documented from an ultrasound report, hospital records or maternal recall, whichever was available, in the given order of preference. The wealth of the family was determined by an index created through a principal component analysis based on household assets [20]. Information on vital status, illnesses (including any hospitalization) along with anthropometric measurements (weight and length) were captured by an independent trained team during their home visits at infant age 1, 3, 6 and 12 months. Caregivers were asked about illness (es) and hospitalization(s) in the 2 weeks preceding the visit. Length was measured using infantometers reading to the nearest 0.1 cm. Exclusivity of breastfeeding was assessed at 1, 3 and 6 months of infant age through a structured questionnaire.

Developmental outcomes were ascertained in the study clinic by trained psychologists using the Bayley Scales of Infant and Toddler Development, 3rd Edition (BSID-III) at 12 months of corrected age [21]. The BSID-III was adapted for use in the study setting. Details of the adaptation have been provided elsewhere [18]. Child stimulation at home was assessed at 12 months of age by trained psychologists using “Pediatric Review of Children’s Environmental Support and Stimulation (PROCESS)” questionnaire [22–24]. PROCESS was created for use with parents of children 2–18 months of age and can be administered in a clinic or in a home setting [22]. It consists of three components: a parent questionnaire, clinical observation, and a toy checklist. The parent questionnaire includes 24 items about the physical environment, household organization, and stimulation practices for development. The 20 observational items focus primarily on the emotional quality of parent-child interactions and the toy checklist consists of 40 items. Total scores are summed across the three sections [22]. Higher scores reflect better stimulation and support to infants. PROCESS scores have been shown to have a good correlation (*r* = 0.84) with the most widely used measure of the household environment i.e., Home Observation for Measurement of the Environment (HOME) scores [23, 24].

### Plan of analyses

All analyses were done using STATA version 16.0 and R version 3.3.3 (2017-03-06). Baseline characteristics were summarized as mean (SD) or proportion. Length-for-age z score (LAZ) was calculated based on the WHO Child Growth Standards, using the zanthro package in STATA [25]. Stunting was defined as LAZ < -2, based on the standard WHO definition [25]. Length measurements were done at 1, 3 and 6 and 12 months of infant age. For this analysis, we preferred to use the length measurements at 6 months instead of 12 months as we wanted to look at the interactions in a cohort approach rather than cross sectionally. Another related premise for adopting such an approach in mid-infancy was that if we could show that linear growth and stimulation at home interacted with each other and influenced each other’s association with neurodevelopment outcomes at 12 months of age, this would provide a reasonable time frame for the caregivers with infants having growth failure to invest in stimulation at home for improving their child’s neurodevelopment. PROCESS scores, reflecting stimulation environment at home, were categorized into three groups: *Low stimulation group* (< Mean-1SD); *moderate stimulation group* (Mean ± 1 SD) and *high stimulation group* (> Mean + 1SD). The mean (SD) PROCESS score was 124 (18).

Neurodevelopmental outcomes consisted of cognitive, motor and language composite scores assessed by BSID-III at 12 months of corrected age. We first measured the association of LAZ at 6 months and PROCESS scores with scores obtained on BSID-III. We selected covariates for adjustment in the model based on their biological plausibility to influence the exposure and the outcomes and purposive selection principle i.e., covariates that brought at least 15% change in the univariate beta-coefficient were included in the multivariable model [26, 27].

We assessed the interaction between LAZ scores at 6 months of age and the PROCESS scores using likelihood ratio test comparing models with and without interaction terms. Analyses were stratified following the identification of a possible interaction. We initially did a screening where a *P*-value for interaction of less than 0.20 was investigated further [28]. The investigation was focussed on examining whether the magnitude of association between LAZ and outcome(s) of interest differed between the subgroups based on PROCESS score categories. Stratified results were presented at differing levels of PROCESS scores (low, moderate and high stimulation). For each of the categories of PROCESS score, we used linear regression with the composite scores for cognition, motor or language as an outcome and LAZ score as the exposure variable. Selection of variables for adjustment in the models was based on biological plausibility and purposive selection principle [26, 27].

Similarly, to assess whether the association between PROCESS scores and neurodevelopmental outcomes was modified by whether the babies were stunted or not, the interaction between the PROCESS score categories and stunting status was assessed using likelihood ratio test comparing models with and without interaction terms. In instances where the *P*-value of interaction was less than 0.20, the analyses were stratified and the effect sizes for the association between PROCESS categories and outcome(s) of interest were presented by the stunting categories. We used generalized additive models (GAM) in the *mgcv package* in R statistical package to depict non-linear associations between PROCESS score, LAZ and outcome scores (composite cognitive, motor and language scores) [29].

### Ethics approval

No ethical approval was required for this secondary data analysis. However, the authors obtained written permission from the principal investigator of the primary trial to use the data for this secondary analysis.

## Results

### Characteristics of the sample

The primary trial enrolled 552 infants of which 516 infants had their neurodevelopment assessment at 12 months of age. The remaining 36 infants either died (*n* = 29) or the families had moved out of the study area (*n* = 7). Baseline characteristics of the 516 infants that were included in this analysis have been presented in Table 1. Supplementary Table 1 presents the comparison of baseline variables between infants with neurodevelopment assessment at 12 months of age and those that did not have the assessment and indicates no statistically significant differences. The infants studied belonged to economically constrained settings as reflected by some of the indicators such as proportion below poverty line (around 23%; national figure of around 15%) and median yearly family income (1316 USD; for some of the developed countries like United States, of around 67,000 USD) [30, 31].

**Table 1:** Baseline characteristics of the infants included in this secondary data analysis (*N* = 516)

Variables	Number (%)
**Household characteristics**	
Yearly family income (in USD); Median (IQR)	1316 (948–2368)
Proportion of families below poverty line	122 (23.7)
**Religion**	
Hindu	423 (81.9)
Muslim	89 (17.3)
Others ^a^	4 (0.8)
**Social class** ^b^	
General	133 (25.8)
Other Backward Class (OBC)	167 (32.4)
Scheduled Caste/Tribe (SC/ST)	216 (41.8)
**Type of family**	
Nuclear	135 (26.2)
Joint	381 (73.8)
**Maternal and paternal characteristics**	
Mean maternal age (years; SD)	23.1 (3.8)
Median years of education of mother (IQR)	5 (0–9)
**Mother’s occupation**	
Home maker	507 (98.3)
Mean father’s age (years; SD)	26.4 (4.7)
Median years of education of father (IQR)	8 (5–12)
**Birth related characteristics**	
**Place of delivery**	
Home	148 (28.7)
Government facility	266 (51.5)
Private facility	102 (19.8)
**Type of delivery**	
Normal vaginal ^c^	511 (99.0)
**Birth order**	
1	191 (37.0)
2–3	232 (45.0)
≥ 4	93 (18.0)
**Parity**	
Primiparous	191 (37.0)
**Infant characteristics**	
**Sex of the baby**	
Male	208 (40.3)
Mean birth weight (grams, SD)	2058.7 (165.3)
Birth weight (range; in grams)	1550–2250
Mean gestational age (weeks, SD)	35.7 (1.9)
Gestational age (range; in weeks)	24–40
Early initiation of breastfeeding (within an hour of birth) present	323 (62.6)
Exclusive breastfeeding at 3 months	250 (48.4)

^a^Others: Christian/Sikh/Jain/Parsi/Zoroastrian/Buddhist/neo Buddhist

^b^General- group that do not qualify for any of the positive discrimination schemes by Government of India (GOI), OBC- term used by the Government of India to classify castes which are socially and educationally disadvantaged, SC/ST- official designations given to groups of historically disadvantaged indigenous people in India

^C^normal unassisted vaginal delivery; USD- United States Dollar; SD- standard deviation; IQR- Inter-quartile range

The mean (SD) composite cognitive, motor and language scores of the sample were 102.1 (11.8), 90.2 (10.4) and 84.9 (9.1) respectively. A total of 52.5% (271/516) of the infants were stunted at 6 months of age. As the exposures of interest i.e., linear growth at 6 months of age and PROCESS scores at 12 months of age were measured after the original intervention (ciKMC) was delivered, we attempted to understand whether ciKMC influenced these exposures. The mean (SD) PROCESS score at 12 months of age was statistically similar in the intervention [123.0 (16.6)] and control [125.0 (16.5)] groups (*P* = 0.16). Further, the mean (SD) LAZ at 6 months of age was also statistically similar in the intervention [− 2.12 (1.04)] and control [− 2.09 (1.06)] groups (*P* = 0.72). The ciKMC intervention did not have any significant association with the cognitive, language and motor outcomes at 12 months of adjusted age [18].

### LAZ, PROCESS score and cognitive outcome

LAZ and PROCESS scores were associated with cognitive scores (Table 2).

**Table 2:** Association of length for age z score (LAZ) and PROCESS score with cognitive, motor and language scores at 12 months of corrected age (*N* = 516)

Variables	Cognitive score	Motor score	Language score
	Adjusted mean difference, b (95% CI) ^a^; *p* value		
LAZ at 6 months	1.78 (0.74, 2.83); *p* = 0.001	2.02 (1.11, 2.94); *p* < 0.001	1.15 (0.37, 1.93); *p* = 0.004
**Stunting status at 6 months**			
Non-stunted	Ref	Ref	Ref
Stunted	−2.99 (−5.11, −0.87); *p* = 0.006	−3.42 (− 5.28, −1.55); *p* < 0.001	−2.53 (−4.11, − 0.95); *p* = 0.002
PROCESS score at 12 months	0.25 (0.18, 0.31); *p* < 0.001	0.16 (0.10, 0.22); *p* < 0.001	0.22 (0.17, 0.27); *p* < 0.001
**PROCESS categories**			
< Mean-1 SD (Low)	Ref	Ref	Ref
Mean ± 1 SD (Moderate)	9.52 (6.47, 12.56); *p* < 0.001	6.60 (3.85, 9.36); *p* < 0.001	7.76 (5.53, 9.99); *p* < 0.001
> Mean + 1SD (High)	12.94 (8.95, 16.95); *p* < 0.001	8.60 (4.98, 12.23); *p* < 0.001	11.82 (8.89, 14.76); *p* < 0.001

^a^Adjusted for wealth quintile, gestational age, birth weight, mother’s education, birth order, exclusive breastfeeding at 3 months, study groups (intervention and control) and hospitalization for severe illness

There was an interaction between LAZ and PROCESS score categories for the cognitive composite score (*P* = 0.08) (Table 3).

**Table 3:** Association between length for age z score and neurodevelopmental outcomes, by PROCESS score categories

Variable	***N*** = 516		
	Adjusted regression coefficient (b) ^**a**^	95% CI	***P***-value
**Cognitive composite score** (*P*-value for interaction between PROCESS score categories and LAZ score = 0.08)			
In low stimulation group (*n* = 72)			
LAZ score	3.63	1.22, 6.03	0.004
In moderate stimulation group (*n* = 367)			
LAZ score	1.41	0.25, 2.56	0.02
In high stimulation group (*n* = 77)			
LAZ score	1.69	−1.15, 4.52	0.24
**Motor composite score** (*P*-value for interaction between PROCESS score categories and LAZ score = 0.03)			
In low stimulation group (*n* = 72)			
LAZ score	4.08	1.69, 6.46	0.001
In moderate stimulation group (*n* = 367)			
LAZ score	1.54	0.50, 2.58	0.004
In high stimulation group (*n* = 77)			
LAZ score	1.05	−1.14, 3.25	0.34
**Language composite score** (*P*-value for interaction between PROCESS score categories and LAZ score = 0.12)			
In Low stimulation group (*n* = 72)			
LAZ score	2.47	0.56, 4.38	0.01
In moderate stimulation group (*n* = 367)			
LAZ score	1.02	0.21, 1.86	0.02
In high stimulation group (*n* = 77)			
LAZ score	0.40	−1.78, 2.58	0.72

^a^Adjusted for wealth quintile, maternal age, maternal education, father’s age, father’s education, parity, birth order, sex of the infant, gestational age, exclusive breastfeeding at 3 months, study groups (intervention and control) and hospitalization for severe illness during infancy; Low stimulation group (PROCESS score; < Mean-1SD); moderate stimulation group (PROCESS score; Mean ± 1 SD) and high stimulation group (PROCESS score; > Mean + 1SD)

*LAZ* Length for age Z score, *PROCESS* Pediatric Review of Children’s Environmental Support and Stimulation, *SD* Standard Deviation

**Fig. 1:**
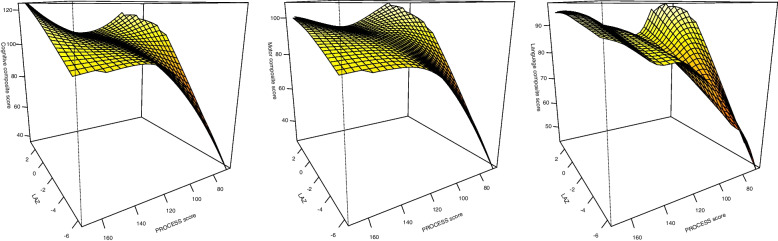
GAM plot depicting the relationship between length-for-age Z score, PROCESS score and cognitive, motor and language composite score

In the low stimulation group, the adjusted regression coefficient (*b* = 3.63, 95% CI; 1.22, 6.03) was substantially higher than in the moderate stimulation group (*b* = 1.41, 95% CI; 0.25, 2.56) and the high stimulation group (*b* = 1.69, 95% CI; − 1.15, 4.52) (Table 3). The GAM plot supports the findings obtained in regression models (Fig. 1). The GAM plot shows that at lower PROCESS scores, the cognitive scores tend to decrease with decrease in LAZ scores whereas at higher PROCESS scores, the relation between LAZ and cognitive score has low variability. Further, with an increase in the PROCESS scores, the cognitive scores increased, more so in those with LAZ less than − 2 SD. An interaction was observed between stunting and PROCESS score categories (Table 4).

**Table 4:** Association between PROCESS score categories and neurodevelopmental outcomes, by stunting status

Variable	***N*** = 516		
	Adjusted regression coefficient (b) ^**a**^	95% CI	***P***-value
**Cognitive composite score** (*P*-value for interaction between PROCESS score categories and stunting categories = 0.17)			
LAZ < -2 (*n* = 271)			
Low stimulation	Ref		
Moderate stimulation	11.09	7.26, 14.92	< 0.001
High stimulation	15.17	9.66, 20.68	< 0.001
LAZ ≥ −2 (*n* = 245)			
Low stimulation	Ref		
Moderate stimulation	6.37	1.77, 10.97	0.007
High stimulation	9.31	3.54, 15.08	0.002
**Motor composite score** (*P*-value for interaction between PROCESS score categories and stunting categories = 0.12)			
LAZ < -2 (*n* = 271)			
Low stimulation	Ref		
Moderate stimulation	8.19	4.49, 11.88	< 0.001
High stimulation	11.76	6.44, 17.06	< 0.001
LAZ ≥ −2 (*n* = 245)			
Low stimulation	Ref		
Moderate stimulation	3.28	−0.53, 7.09	0.09
High stimulation	4.24	−0.54, 9.03	0.08
**Language composite score** (*P*-value for interaction between PROCESS score categories and stunting categories = 0.05)			
LAZ < -2 (*n* = 271)			
Low stimulation	Ref		
Moderate stimulation	9.26	6.58, 11.95	< 0.001
High stimulation	14.19	10.32, 18.05	< 0.001
LAZ ≥ −2 (*n* = 245)			
Low stimulation	Ref		
Moderate stimulation	4.29	0.82, 7.75	0.02
High stimulation	7.48	3.13, 11.83	0.001

^a^Adjusted for wealth quintile, maternal age, maternal education, father’s age, father’s education, parity, birth order, sex of the infant, gestational age, exclusive breastfeeding at 3 months, study groups (intervention and control) and hospitalization for severe illness during infancy; Low stimulation (PROCESS score; < Mean-1SD); moderate stimulation (PROCESS score; Mean ± 1 SD) and high stimulation (PROCESS score; > Mean + 1SD)

*LAZ* Length for age Z score, *PROCESS* Pediatric Review of Children’s Environmental Support and Stimulation, SD Standard Deviation

In both stunted and non-stunted infants, PROCESS scores were associated with cognitive scores with a clear dose response relationship (Table 4). The adjusted regression coefficient was comparatively higher in stunted infants.

### LAZ, PROCESS score and motor outcome

LAZ and PROCESS scores were associated with motor scores (Table 2). There was an interaction between LAZ and PROCESS score categories for the motor composite score (*P* = 0.03) (Table 3). In the low stimulation group, the adjusted regression coefficient (*b* = 4.08, 95% CI; 1.69, 6.46) was higher than in the moderate stimulation (*b* = 1.54, 95% CI; 0.50, 2.58) and the high stimulation group (*b* = 1.05, 95% CI; − 1.14, 3.25) (Table 3). The GAM plot confirmed the findings obtained in regression models (Fig. 1). An interaction was observed between stunting and PROCESS score categories (Table 4). In stunted infants, PROCESS scores were associated with motor composite scores with a dose response relationship. In non-stunted infants, the adjusted regression coefficient was comparatively lower and did not reach statistical significance.

### LAZ, PROCESS score and language outcome

LAZ and PROCESS scores were associated with language scores (Table 2). A potentially relevant interaction (*P* = 0.12) was observed between LAZ and PROCESS score categories (Table 3). In the low stimulation group, the adjusted regression coefficient (*b* = 2.47, 95% CI; 0.56, 4.38) was substantially higher than in the moderate stimulation (*b* = 1.02, 95% CI; 0.21, 1.86) and high stimulation group (*b* = 0.40, 95% CI; − 1.78, 2.58) (Table 3). The GAM plot confirmed the findings obtained in regression models (Fig. 1). An interaction was observed between stunting and PROCESS score categories (*P* = 0.05) (Table 4). In both stunted and non-stunted infants, PROCESS scores were associated with language scores with a distinct dose response relationship. The adjusted regression coefficient was comparatively higher in stunted infants.

## Discussion

The current analyses aimed at providing answers to questions with programmatic implications, specifically whether within a setting with socio-economic constraints, a moderate to high-quality home environment can alleviate the risk of low development scores in LBW infants with linear growth deficits. We observed a weakening of the association between growth deficits and negative neurodevelopment outcome with increase in stimulation and nurturance at home. Additionally, we also observed that while stimulation at home was associated with neurodevelopmental outcomes in both stunted and non-stunted infants, the association was stronger in stunted than non-stunted infants.

Our findings corroborate the studies done in Bangladesh, Vietnam and in African settings (Burkina Faso, Malawi and Ghana) where the authors noted that among non-low birth weight children, a nurturant home environment attenuated the association between linear growth and neurodevelopmental outcomes [13–15]. However, findings contrast with the results of the studies among the Malaysian and Jamaican children where no significant influence of home environment quality on the association between LAZ status and cognitive outcomes was noted. The observed difference might be due to fairly smaller sample sizes in these studies, thereby reducing the power to detect significant interactions [16, 17].

There is lack of consensus with regards to the consideration of *P*-value to indicate presence of an interaction. While some investigators propose to adhere to the conventional *P*-value of < 0.05, others suggest that usually the power to test for interactions is low in many epidemiologic studies and therefore, testing for interaction tests based solely on *P*-value of < 0.05 may be misleading and could probably miss out important effect modifications [28, 32–34]. Based on this consideration, the suggestion is to increase the type 1 error rate to 20% while assessing tests of interaction [28]. Some researchers argue that consideration of a *P*-value for interaction tests is a part of the entire spectrum of information to be utilized in the assessment of effect modification and other components should be considered such as stratum-specific measures and prior biological knowledge [35, 36]. In our study, we considered a *P*-value of less than 0.20 to further investigate for potential interaction. Subsequently, we placed emphasis on the magnitude of effect size within the subgroups and attempted to make careful interpretations. Our findings were also supported by the GAM plots that depicted non-linear relationships between LAZ, PROCESS score and neurodevelopment outcomes.

There are strengths and limitations of this secondary data analyses. The data utilized is from a robust and well conducted randomized controlled trial with very low attrition. The measurements, including the anthropometry, and outcome data were collected by trained and standardized study team. One of the limitations is that the study lacks reliable data on gestational age. Weight was measured within 72 hours of birth by trained study team and inclusion of infants with weight between 1500 and 2250 g meant that these infants would be either preterm or term small for gestational age. Therefore, the findings could be extended only to a specific population of LBW infants i.e., stable late preterm or term small for gestational age (SGA) infants. There could also be a possibility that in babies with poorer linear growth or smaller attained length at 6 months of age or rather the factors that lead to such growth faltering may lead to poorer home stimulation which is measured 6 months later by PROCESS. Measurement of home stimulation at one time point only i.e., at 12 months is also a limitation. We also acknowledge that this being an observational study, the results may be affected by unmeasured confounding.

Our findings support the promotion of stimulation to LBW infants in order to offset the negative effect of growth faltering on neurodevelopmental outcomes. It is likely that every child will benefit from this strategy and therefore, future studies should test this approach in normal/non-high-risk children as well. Our findings indicate that through focusing only on nutrition for growth, we may miss to capitalize the important developmental effects of early child stimulation and responsive caregiving. The findings call for a comprehensive approach with nutrition and nurturing care at the forefront. This approach underlies the comprehensive framework of Nurturing Care that incorporates health, nutrition, responsive caregiving, opportunities for early learning, and child protection as a way to help children not only survive but also thrive [37].

## Conclusion

The findings suggest that a moderate to high-quality stimulation at home may alleviate the risk of poor development scores in LBW infants with linear growth deficits. Early child stimulation may particularly be beneficial for LBW infants with linear growth deficits/stunting. Efforts for improving child development should be comprehensive with promotion of adequate nutrition and optimal nurturing care as integral components.

## Supplementary Information

**Additional file 1: Supplementary Table 1.** Comparison of baseline characteristics of the infants with neurodevelopment data at 12 months (*N* = 516) and those that did not have (*N* = 36).

## Data Availability

The dataset analysed in the present study is publicly available at: https://figshare.com/s/c932d11ff5101e2268bf.

## Abbreviations

LBW: Low birth weight
PROCESS: Pediatric Review of Children’s Environmental Support and Stimulation tool
LAZ: Length for age Z score
RCT: Randomized controlled trial
CiKMC: Community initiated Kangaroo Mother Care
HBPNC: Home Based Post Natal Care
BSID-III: Bayley Scales of Infant and Toddler Development, 3rd Edition
HOME: Home Observation for Measurement of the Environment
GAM: Generalized additive models
SGA: Small for gestational age
AGA: Appropriate for gestational age
