# Supplementary material for: Early child stimulation, linear growth and neurodevelopment in low birth weight infants
Source: BMC Pediatr. 2022 Oct 8;22:586. doi: 10.1186/s12887-022-03579-6 (PMC9547474; doi:10.1186/s12887-022-03579-6)
Supplement: Supplementary file 1 — Additional file 1: Supplementary Table 1. Comparison of baseline characteristics of the infants with neurodevelopment data at 12 months (N = 516) and those that did not have (N = 36). [file 12887_2022_3579_MOESM1_ESM.doc]

**Supplementary Table 1. Comparison of baseline characteristics of the infants with neurodevelopment data at 12 months (N=516) and those that did not have (N=36)**

| **Variables** | **N=516**  **n (%)** | **N=36**  **n (%)** |
| --- | --- | --- |
| **HOUSEHOLD CHARACTERISTICS** | |  |
| Yearly family income (in USD); Median (IQR) | 1316 (948-2368) | 1207 (857-1929) |
| Proportion of families below poverty line | 122 (23.7) | 4 (11.1) |
| **Religion**  Hindu  Muslim  Others ^a^ | 423 (81.9)  89 (17.3)  4 (0.8) | 27 (75.0)  9 (25.0)  0 (0.0) |
| **Social class** ^b^  General  Other Backward Class (OBC)  Scheduled Caste/Tribe (SC/ST) | 133 (25.8)  167 (32.4)  216 (41.8) | 4 (11.1)  14 (38.9)  18 (50.0) |
| **Type of family**  Nuclear  Joint | 135 (26.2)  381 (73.8) | 5 (13.9)  31 (86.1) |
| **MATERNAL AND PATERNAL CHARACTERISTICS** | |  |
| Mean maternal age (years; SD) | 23.1 (3.8) | 22.8 (3.4) |
| Median years of education of mother (IQR) | 5 (0-9) | 5 (0-9) |
| **Mother’s occupation**  Home maker | 507 (98.3) | 35 (97.2) |
| Mean father’s age (years; SD) | 26.4 (4.7) | 26.2 (4.9) |
| Median years of education of father (IQR) | 8 (5-12) | 8 (5-10) |
| **BIRTH RELATED CHARACTERISTICS** | |  |
| **Place of delivery**  Home  Government facility  Private facility | 148 (28.7)  266 (51.5)  102 (19.8) | 12 (33.3)  17 (47.2)  7 (19.5) |
| **Type of delivery**  Normal vaginal ^c^ | 511 (99.0) | 35 (97.2) |
| **Birth order**  1  2-3  ≥4 | 191 (37.0)  232 (45.0)  93 (18.0) | 15 (41.7)  12 (33.3)  9 (25.0) |
| **Parity**  Primiparous | 191 (37.0) | 15 (41.7) |
| **INFANT CHARACTERISTICS** | |  |
| **Sex of the baby**  Male | 208 (40.3) | 21 (58.3) |
| Mean birth weight (grams, SD) | 2058.7 (165.3) | 2058.5 (192.1) |
| Mean gestational age (weeks, SD) | 35.7 (1.9) | 35.5 (2.0) |
| Early initiation of breastfeeding present | 323 (62.6) | 18 (50.0) |
| Exclusive breastfeeding at 3 months | 250 (48.4) | 20 (55.5) |

^a^ Others: Christian/Sikh/Jain/Parsi/Zoroastrian/Buddhist/neo Buddhist; ^b^ General- group that do not qualify for any of the positive discrimination schemes by Government of India (GOI), OBC- term used by the Government of India to classify castes which are socially and educationally disadvantaged, SC/ST- official designations given to groups of historically disadvantaged indigenous people in India; ^C^ normal unassisted vaginal delivery; USD- United States Dollar; SD- standard deviation; IQR- Inter-quartile range
